# Urogenital Infections Among Women Attending Mwingi Hospital, Kitui County, Kenya: Safeguarding Antibiotics Through Microbiological Diagnosis

**DOI:** 10.24248/eahrj.v8i1.764

**Published:** 2024-03-28

**Authors:** Christine Musungi Mbuvi, Boniface Nzioki Musila, Anthony Kebira Nyamache

**Affiliations:** a Dept of Population, Reproductive Health & Community Resource Management, Kenyatta University; b Dept of Obstetrics & Gynecology, Kenyatta University; c Dept of Biochemistry Microbiology & Biotechnology, Kenyatta University

## Abstract

**Background:**

Urogenital infections pose a considerable public health threat, as almost half of women will experience urinary and reproductive system infections at some point in their lives. However, the urogenital infection burden is often not clear in some regions. Nevertheless, the misuse of antimicrobial agents, including self-prescription, has increased widespread antimicrobial resistance, limiting treatment benefits. Therefore, this study aimed to identify the various urogenital infections, associated risk factors, and profile the bacterial isolates, and assess their antibiotic resistance among women attending Mwingi Hospital.

**Methods:**

A cross-sectional study was conducted on 322 women aged between the ages of 15 to 44 years. Urine and high vaginal swabs were collected from all participants and analyzed within 6 hours. Microscopic examination on wet mounts was done, bacterial isolation was done and those with significant growth were confirmed and subjected to antimicrobial susceptibility testing using specific media. Descriptive statistics were used in expressing the infection frequencies and antimicrobial resistance. Odds ratios were used to determine the risk of urogenital infection. The level of significance was considered at a P value of less than 0.05.

**Results:**

Among the 322 women, 45.3% (146) had a urogenital infection, with bacteria being the primary cause (26.4%). The infections included UTI (22.7%), *Candidiasis* (15.2%), *Trichomoniasis* (3.7%), *Gonorrhea* (2.5%), and *Bacterial vaginitis* (1.2%). Antibiotic use was 32.9%, with only 2.8% receiving a microbiological diagnosis before antibiotic use. The overall antibiotic resistance was 53%, with the lowest resistance observed against penicillin and combinations (31.4%) and 3rd Cephalosporins (39.4%). The highest resistance was observed against nalidixic acid (74.8%) and cotrimoxazole (62.6%).

**Conclusion:**

Women attending Mwingi Hospital are commonly affected by various urogenital infections. Antibiotic use without microbiological diagnosis was observed. Among the antibiotics tested, 3^rd^ generation cephalosporins and penicillin combination agents were noted as the most effective in treating bacterial urogenital infections, while nalidixic acid and cotrimoxazole were ineffective. Improved diagnosis and targeted treatments are necessary to prevent further development of antibiotic resistance.

## BACKGROUND

Urogenital infections in women of reproductive age (15 to 44 years) remain a significant cause of morbidity, especially in developing nations.^1^ These infections develop when microorganisms infect the reproductive or urinary tract organs. Many microorganisms, including bacteria, protozoa, and fungi, cause urogenital infections. Globally, urinary tract infections (UTIs) are reported by 50% of women at some point in their lives.^2^ On average, over one million new reproductive organ infections are reported each day.^1^ In Kenya, the prevalence of UTI is 27.6%, ^3^ while that of *Trichomoniasis* and *Bacterial vaginosis* are 7.4% and 19.3% respectively.^4^

The diagnosis of urogenital infection is achieved by determining the specific microorganism causing the infection through microbiological diagnosis.^5^ The World Health Organization recommends culture and antimicrobial testing as the best options for diagnosing and managing bacterial and fungal infections.^6^ In healthcare facilities where microbiological testing is not available for the diagnosis of urogenital infections, treatment is offered based on clinical presentations and routine urinalysis.^7,8^ Thus, various antimicrobials are blindly offered, leading to their overuse and misuse. A study conducted in Kenya reported that out of 3590 hospitalized patients, 46.7% were on antimicrobials, with only 0.1% being treated based on microbiological tests.^9^ This has resulted in the emergence of antibiotic resistant bacteria and treatment failure, which are of great public health concern.^8^

Studies have shown high rates of antibiotic resistance, even in recently approved antibiotics. In a study involving women of reproductive age at Pumwani Hospital 49% overall antibiotic resistance was detected against 2^nd^ and 3^rd^ generation of Cephalosporins, 2^nd^ generation of quinolones, Sulfamethoxazole-Trimethoprim, Nitrofurantoin, and Penicillin & a combination.^10^

To address these challenges, advocacy on antimicrobial resistance as a keystone of the Global Action Plan on antimicrobial resistance has been executed. This plan has been endorsed by the World Organization for Animal Health delegates, the World Health Assembly, and the Food and Agriculture Organization.^11^ From a local perspective, Kenya launched a global antimicrobial strategy through a partnership project that was started in 2009 and completed the analysis of the antimicrobial resistance (AMR) situation in 2011. Despite these important milestones in improving awareness and understanding of antimicrobial resistance in Kenya, the cases of escalating levels of antimicrobial resistance have continued to increase over time.^12^ It is therefore against the above challenge, that this study investigated and described the various urogenital infections through microbiological diagnosis, their risk factors, and antibiotic resistance among women attending Mwingi Level IV Hospital in Kitui County, Kenya.

## MATERIALS AND METHODS

### Study Area

The study was carried out at Mwingi Level IV Hospital during the period between August 2021 and May 2022. Mwingi Hospital is a sub-county referral facility providing comprehensive obstetric care for both outpatient and inpatient treatment outlets. The hospital is located within Mwingi central constituency, Kitui County, Kenya.^13^

### Study Population

The study targeted women aged between the ages of 15 to 44 years seeking medical care at the outpatient clinics of Mwingi level IV hospital. Three hundred and twenty-two (322) women consented to the study's protocols and were consecutively recruited into this study

### Sample Collection

Sterile mid-stream urine and high vaginal swabs (swabs from the vaginal vault) were collected from consenting study participants and sent to the laboratory within 6 hours for examination. This study was ethically approved by Kenyatta University's ethical scientific committee before execution.

### Urinalysis

A standard dipstick test was conducted on all urine samples to assess the semi-quantitative levels of leukocytes, nitrites, blood, specific gravity, pH, and proteins in urine. The urine sediments were examined under 10x and 40x microscopy lenses to identify any white blood cells, *Trichomonas Vaginalis,* and bacteria.^14^ A positive result for leukocyte esterase and/or nitrites following the urine dipstick test, the presence of more than six white blood cells per 40x microscope lens, and the detection of bacteria and fungi in urine were all indicative of symptomatic infections then confirmed with culture.^15^ The presence of *Trichomonas vaginalis* in urine sediments confirmed trichomoniasis infection.^16^

### High Vaginal Swabs Microscopy

Examination of high vaginal swabs (swabs from the vaginal vault) was performed using 10x and 40x microscope lenses to identify any *Trichomonas vaginalis*, white blood cells, fungi, and bacteria that were symptomatic of an infection.^16^ Bacteria and fungi were confirmed using culture and biochemical tests. While the presence of *Trichomonas vaginalis* in vaginal swabs confirmed trichomoniasis infection.^16^

### Culture

Using a sterile calibrated wire loop, about 10ul urine samples were inoculated onto CLED, Blood agar, and chocolate agar.^17^ High vaginal swabs were inoculated into chocolate agar, MacConkey agar, and blood agar and incubated at 5% to10% carbon dioxide at 37°C for 18 to 24 hours. Bacterial isolates were identified based on cultural characteristics. These presumptive suspected bacterial and fungal species isolates were confirmed using Gram-stain and biochemical tests.^16^

### Antimicrobial Susceptibility Testing

Known 0.5 McFarland's standards pure bacteria isolates were inoculated on Muller-Hinton while Muller-Hinton chocolate agar was used in suspected *Neisseria* spp. Antibiotic disks were evenly distributed on the agar surface and incubated at 37°C for 20–24 hours.^16^ The diameter of zones of inhibition was measured and compared to those of standards for antimicrobial susceptibility testing according to the Clinical and Laboratory Standards Institute-2020.^16,18,19^

### Statistical Analysis

All data were analyzed using the statistical software IBM SPSS version 25. Descriptive statistics were in expressing frequencies in urogenital infections and antibiotic resistance. Odds ratios were used to analyze the risk of developing a urogenital infection while regression analysis was used to determine the risk factors associated with urogenital infections. The *P* value of less than .05 was used as the level of significance.

### Ethical Consideration

The ethical approval was obtained from the Kenyatta University ethics review committee; ethics approval number PKU/2313/11452, before its execution. In addition, permission to be allowed to conduct this study at Mwingi Level IV hospital was also granted by hospital management. The study was conducted according to the Declaration of Helsinki and data for each participant was kept confidential.

## RESULTS

### Participants Demographics

This study included a total of 322 women aged between 15 and 44 years who were seeking healthcare at the outpatient clinics of Mwingi Hospital. Of the women, 38.8% were aged between 15-24 years, 37.6% were between 25-34, and 23.6% were aged between 35-44 years. 234 (72.7%) women were married and 88 (27.4%) were single, divorced, separated, or widowed. (Table 1)

**TABLE 1:** Profile of Study Participants (Odds Ratio Analysis)

Variables	Infection	No infection	Odds ratio	95% CI
Age				
15 – 24	18.3% (59)	20.5% (66)	1.107	0.706–1.736
25 – 34	16.5% (53)	21.1% (68)	1.127	0.716–1.774
35 – 44	10.9% (35)	12.7% (41)	0.979	0.584–1.641
Marital status				
Married	33.9% (109)	38.8% (125)	1.147	0.700–1.188
Single/widow/divorced	11.5% (38)	15.5% (50)	0.940	0.532–1.428
Family planning method				
Hormonal	13.4% (43)	13.0% (42)	1.309	0.797–2.151
IUD	2.5% (8)	5.9% (19)	0.473	0.201–1.113
Barrier	1.2% (4)	2.17% (7)	0.671	0.193–2.340
None	28.6% (92)	33.2% (107)	1.063	0.677–1.670
Antibiotic use				
Within 3 months	19.9% (64)	13.4% (43)	2.367	1.473–3.803
More than 3 months	25.8% (83)	40.9% (132)	0.422	0.263–0.679
Pregnancy				
Pregnant	19.6% (63)	20.0% (65)	1.269	0.811–1.987
Not pregnant	26% (84)	34.1% (110)	0.788	0.503–1.233
Sexual activities				
Sexually active	39.1% (126)	45% (145)	1.241	0.677–2.277
Not sexually active	6.5% (21)	9.3% (30)	0.806	0.439–1.478
Material of undergarment				
Cotton	17.7% (57)	24.8% (80)	0.752	0.482–1.174
Non-cotton	28% (90)	29.5% (95)	1.330	0.852–2.076
Sanitary change				
Within 3 hours	0.3% (1)	0.6% (2)	0.592	0.053–6.6
After 3 hours	45.3% (146)	53.7% (173)	1.688	0.152–18.803
Sanitary type				
Pad & tampon	35.7% (115)	37.6% (121)	1.604	0.967–2.227
Different materials	9.9% (32)	16.8% (54)	0.624	0.376–1.034
Sanitary change				
Within 3 hours	0.3% (1)	0.6% (2)	0.592	0.053–6.6
After 3 hours	45.3% (146)	53.7% (173)	1.688	0.152–18.803
Chronic infections				
HIV	3.7% (12)	4.0% (13)	1.108	0.489–2.508
Diabetes	1.8% (6)	1.2% (4)	1.819	0.503–6.573
None	40.0% (129)	49.1% (158)	0.771	0.382–1.557

### Urogenital Infections Among Women Attending Mwingi Hospital

The overall prevalence of urogenital infections was 45.3% obtained from 322 women, including urinary tract infections (UTI) in 22.7%, candidiasis in 15.2%, trichomoniasis in 3.7%, gonorrhea in 2.5%, and bacterial vaginitis in 1.2 % of women attending Mwingi Hospital.

### Risk Factors of Urogenital Infections

Among the 322 women, 106 (32.9%) reported having used antibiotics within three months at the time of this study, and 97.2% received antibiotic treatment without a microbiological laboratory diagnosis. The prevalence of urogenital infections was 59.4% (n=63) among antibiotic users, who were at increased risk of urogenital infection 2.37 (95%: CI 1.47–3.80) compared to those who never used antibiotics within the three months.

Urogenital infection did not significantly vary with age (OR 1.1; 95%: CI 0.72-1.77), marital status (OR 1.15; 95%: CI 0.70-1.19), use of family planning methods (OR 1.31; 95%: CI 0.79-2.15), pregnancy status (OR=1.27; 95%: CI 0.81-1.99), women's status of sexual activities (OR=1.24; 95%: CI 0.68-2.28), HIV status (OR=1.11; 95%: CI 0.49-2.51), and Diabetes (OR=1.82;95%, CI 0.503-6.573). (Table 2)

**TABLE 2:** Risk Factors of Urogenital Infection (Multiple Regression Analysis)

Variable	Odds ratio	95% CI	*P value*
Age	1.107	0.706–1.736	.340
Marital status	1.147	0.700–1.188	.923
Family planning method	1.309	0.797–2.151	.562
Chronic illness	1.819	0.503–6.573	.889
Pregnancy	1.269	0.811–1.987	.310
Sexually activities	1.241	0.677–2.277	.618
Antibiotic use	2.367	1.473–3.803	.030
Sanitary type	1.604	0.967–2.227	.609
Frequency of sanitary change	1.688	0.152–18.803	.255
Material of undergarment	1.604	0.967–2.227	.843

Adjusted R2 = 0 .026

### Bacterial Isolates and Antibiotic Resistance

All the 322 women provided urine and high vaginal swabs for examination. However, a total of 196 were cultured having met the criteria for culture. These included 67 high vaginal swabs and 128 urine samples. Of these, 24 high vaginal swabs and 59 urine samples gave a total of 83 culture plates that had significant bacterial growth. The majority of the bacterial isolates were Gram-positives 42 (50.6%) and gram-negatives were 41 (49.4%). *Staphylococcus aureus* (39.8%) was the most prevalent isolate, *E. coli* (24.2%), *N. gonorrhea* (10.8%), *Enterococcus* spp (9.6%), *Pseudomonas aeruginosa* (6.0%), *Klebsiella* spp (3.6%), *proteus* spp (2.4%), *Enterobacter* spp (2.4%) and *Streptococcus* spp (1.2%) was the least isolated. Bacterial resistance was highest against Nalidixic acid (74.80), Levofloxacin (67.55%), Clarithromycin (62.90%), and Cotrimoxazole (62.60%), while the lowest resistance was observed for Piperacillin/Tazobactam (16.90%), Ceftazidime (36.10%) and Cefixime (36.35%). Gram-negative bacteria were the most resistant (55.9%), compared to gram-positive bacteria (43.8%). More details on antibiotic resistance are shown in Table 3.

**TABLE 3:** Antibiotic Resistance to Urogenital Infection

Antibiotic class	Antibiotics	Gram-positive	Gram-negative	Average %	Overall resistance
Penicillin & combinations	Piperacillin/Tazobactam	16.7	17.1	16.9	31.4
	Amoxycillin/Clavulanic	33.3	58.5	45.9	
3rd Cephalosporins	Ceftazidime	38.1	34.1	36.1	39.4
	Cefixime	19	53.7	36.4	
	Ceftriaxone	50	41.5	45.8	
Oxazolidinones	Linezolid	31	80.5	55.8	55.6
Macrolides	Clarithromycin	42.9	82.9	62.9	56.2
	Erythromycin	47.6	51.2	49.4	
2nd Quinolones	Ciprofloxacin	54.8	34.1	44.5	56
	Levofloxacin	59.5	75.6	67.6	
1st Quinolones	Nalidixic acid	66.7	82.9	74.8	74.8
Sulfamethoxazole	Cotrimoxazole	66.7	58.5	62.6	62.6
	Average resistance	46.3	59.4	53	

## DISCUSSION

### Prevalence of Urogenital Infections

The most frequent urogenital infections among women at Mwingi Hospital were UTIs. This prevalence is within the 13% to 33% range for global UTI prevalence,^20^ albeit marginally lower than the 32.2% reported in Uganda,^21^ and the 27.6% reported in Kenya.^3^

Vaginal candidiasis prevalence in this study was higher than some of the studies done earlier, Germany reported 5.3%,^22^ and Kenya reported 3%.^23^ The incidence of candidiasis depends on individual personal lifestyle and hygiene.^24^ Sexually transmitted infection (STI) prevalence among women at Mwingi Hospital is 2.5%, while it is 9.02% in Kilifi.^4^

### Risk Factors of Urogenital Infections

This study observed that antibiotic use had a significant association with to risk of contracting urogenital infections. The regression *P* value (*P*=.03) was significant. Overuse of antibiotics has led to the emergence of antibiotic-resistant bacteria and treatment failures.^8^ In addition, the woman's natural flora is typically changed as a result of excessive use of antibiotics that in turn increases the susceptibility to infections.^24^

Women aged 15 to 34 years were at 1.1 times higher risk of developing a urogenital infection compared to those aged between 35 to 44 years. This contrasts favorably with three studies, where women aged between 18 to 35 years had higher odds of developing a urogenital infection.^4,25,26^

Compared to other forms of contraception, hormonal contraceptive users had the highest overall prevalence of urogenital infections. There was an increased risk of urogenital infections among hormonal contraception users, which is in line with other investigations.^27,28^ Oestrogen or progestins which are components of hormonal birth control, lower serum levels of oestrogen and progesterone. A higher vaginal PH value is linked to low levels of these hormones, which raises the risk of vaginal infections.^24^

The body's immune system against microorganisms is weakened by chronic illnesses such as HIV and diabetes, which may increase the risk of urogenital infections.^29^ A person with diabetes has a higher risk of developing infections, and this can sometimes make treatment more challenging since higher serum sugar levels promote the growth of yeast cells.^30^

Compared to women who wore cotton undergarments, women who used non-cotton undergarments had a higher prevalence and increased risk of urogenital infections, and those who never changed their sanitary items within three hours. According to earlier research, maintaining excellent hygiene, like the use of absorbent cotton undergarments, undergarments at least once daily, and wiping from the anterior to the posterior after long or short calls, can reduce the incidence of urogenital infection.^24,27^

Pregnancy or marital status didn't have a significant association with the risk of contracting urogenital infections. However, infections were present in 1 out of 5 pregnant women with a higher risk of contracting a urogenital infection than non-pregnant women. This prevalence was higher compared to the 2.5% reported among pregnant women in Nairobi.^31^ A urinary tract infection will occur in roughly 40% of pregnant women in rural Kenya at some stage during their pregnancy.^28^ Despite the important impact that good personal hygiene plays in the development of bacteriuria, hormonal changes during pregnancy might also raise the risk of vaginal infections due to changes in PH in the vagina as a result.^21^

### Antibiotic resistance

Gram-positive bacteria made up slightly more than gram-negative in this study. The major bacterial isolates were E. coli and *Staphylococcus aureus*. Studies have reported *E. coli* and *Staphylococcus aureus* as the two frequent bacteriuria isolates.^10,21^ Gram-negative bacteria generally showed more resistance to the antimicrobials evaluated in this investigation than gram-positive isolates did. This finding concurs with previous studies that have been conducted in Kenya and elsewhere.^10,21,28^ Due to bacterial distinct cell walls, antibiotic resistance is higher in Gram-negative bacteria than in Gram-positive bacteria. Gram-negative bacteria have multi-layered cell walls, whereas gram-positive bacteria have a thick peptidoglycan layer that absorbs antibiotics more readily.^32,33^

Geographical settings and individual lifestyles affect susceptibility patterns differently. Slightly above a half of antibiotic resistance was obtained in this study. Third-generation cephalosporin resistance was observed although at the least. These findings were similar to two other studies conducted in Pumwani and rural Kenya.^10,28^ Since third-generation cephalosporins have been widely used in Kenya, organisms have gradually developed a variety of resistance mechanisms.^10^

In this study, Penicillins and combinations were generally susceptible; Piperacillin/Tazobactam was less resisted than Amoxycillin/Clavulanic. Resistance to macrolides was moderate; Clarithromycin was more resisted than Erythromycin.

Resistance to quinolones was above average; Levofloxacin showed higher resistance rates than Ciprofloxacin. The major contributors to the global increase in antimicrobial drug resistance are the aimless application of antibiotics to upgrade growth in veterinary medicine, over-the-counter selling of antibiotics, and non-compliance to the directed timelines of treatment.^11^ Studies in Kenya have reported wide consumption of antibiotic residues in commercial meat which may lead to antibiotic resistance in consumers. A study in Kiambu obtained a 41.6% prevalence of antibiotic residues in pork meat.^34^ Analysis among smallholder farms in Kenya obtained a 24% positivity rate of antibiotic residues in commercial milk. The antibiotic detected constituted tetracyclines, beta-lactams, and sulfonamides.^35^

In Nyeri, 23.4% had self-medicated with antibiotics, among which 60.6% took the antibiotics without a diagnosis.^36^ Self-medication to antibiotics is a driver to increase antibiotic resistance since there is no focus on the antibiotic drug that matches a bacteria's susceptibility. Antibiotic resistance after antibiotic therapy is common and is not a result of bacterial mutation but rather reinfection with a different bacterial strain resistant to the previous antibiotic.^8^ An estimated 4.95 million deaths occur annually due to antimicrobial related deaths and without proper interventions, antimicrobial resistance disease-related deaths are expected to rise to 10 million annually by 2025. Concerted and coordinated global action is necessary to contain antimicrobial resistance.^37^

### Limitations to the study

Since this study was limited to women, its findings may not be generalized to the entire population. In addition, based that this was a hospital-based Cross-sectional study, it may not be used to represent Kitui County.

## CONCLUSION

This study observed that various urogenital infections prevail among women attending Mwingi Hospital. The infections constituted; UTI, candidiasis, trichomoniasis, gonorrhea, and bacterial vaginitis of women attending Mwingi Hospital. Women aged (15-34 years), those using the hormonal family planning method, those with chronic diseases, those who are pregnant, sexually active, and those with poor personal hygiene had an increased risk of urogenital infection with no significant association with contracting a urogenital infection. For the observed rate of antibiotic use during the study, most received antibiotic treatment without a microbiological laboratory diagnosis. The least resistance was observed against penicillin and combinations and 3^rd^ Cephalosporins. The highest resistance was observed against nalidixic acid and cotrimoxazole.

### Recommendation

Multifaceted interventions to embrace microbiological laboratory diagnosis for targeted treatments of urogenital infections at Mwingi Hospital. Development and advocacy of strategies for reducing risk factors for urogenital infections among women attending Mwingi Hospital.
